# Development of Autoranging Functionality to Enhance the Sensitivity of Helical Capacitance Level Sensors via Neural Networks

**DOI:** 10.3390/s26175464

**Published:** 2026-08-28

**Authors:** Jayalaxmi Rajesh Hanni, Santhosh Krishnan Venkata

**Affiliations:** 1 Manipal Institute of Technology Bengaluru, Manipal Academy of Higher Education, Manipal, Udupi 576104, Karnataka, India; jayalaxmi.hanni@manipal.edu; 2 Manipal Institute of Technology, Manipal Academy of Higher Education, Manipal, Udupi 576104, Karnataka, India

**Keywords:** autorange, capacitance level sensors, neural networks, sensitivity analysis

## Abstract

**Highlights:**

- This study developed an ANN-based intelligent autoranging technique for adaptive sensor calibration.
- The method enhanced sensor sensitivity across user defined measurement ranges without recalibration.
- The approach was validated using a capacitance level sensor with a 60 cm helical electrode.
- The full-scale sensitivity improved from 4.9 V/m to 58 V/m, enabling accurate process measurements.

**Abstract:**

Sensitivity is a critical performance parameter of sensors used in industrial process measurement systems. Conventional sensors are typically calibrated for a predefined operating range, resulting in reduced measurement sensitivity and accuracy when users require measurements within alternate subranges of the sensor’s operating band. To address this limitation, this paper proposes an intelligent autoranging technique that dynamically enhances sensor sensitivity across user-defined measurement ranges without the need for manual recalibration. The proposed approach integrates an adaptive calibration framework based on artificial neural network (ANN) algorithms to automatically adjust the sensor response for different operating intervals. The methodology is implemented and experimentally validated using a capacitance level sensor (CLS) incorporating a 60 cm helical electrode structure for liquid-level measurement. The experiments were conducted using water as the test liquid under controlled laboratory conditions. The sensor capacitance, varying from 0.929 nF to 421 μF over a liquid-level range of 0 to 60 cm with a resolution of 0.1 cm, is converted into a measurable voltage signal ranging from 0.010939 V to 2.953 V through a dedicated signal conversion and conditioning circuit. The experimental results demonstrate that the proposed ANN-based autoranging strategy significantly improves sensor performance by increasing the full-scale sensitivity from 4.9 V/m to 58 V/m across different user-selected measurement ranges. The developed technique offers a flexible and intelligent solution for enhancing sensor sensitivity and adaptability in process industry applications, enabling accurate measurements over varying operating ranges without additional calibration procedures.

## 1. Introduction

Capacitance level is among the essential process parameters including flow, pressure, and temperature and requires precise measurement and control in many applications. Industries rely on accurate level measurements for processes involving dairy products, pharmaceuticals, dyes, and liquid levels in tanks [1]. Various sensors, including conductive probes; floats; radar; and optical, ultrasonic, pressure, and capacitance-level sensors (CLSs), are used because of their low cost, high repeatability, easy installation, and user-friendly operation [2]. The capacitance level measurement technique works on the principle of a capacitor [3]. Optical devices using interferometer principles are used to measure the level of liquid in the range of 0–45 cm, with a sensitivity of approximately −0.016 dBm/mm [4]. Similar studies were presented in [5] to measure cryogenic liquid levels via Gaussian beams for a lower range of measurements. In [6], a sensor design utilizing the twisted coupling technique of two optical fibres was used in liquid-level sensors to measure depths up to 125 mm with a sensitivity of 8.03 nW/mm.

A polymer optical fibre Bragg grating is used to measure the liquid level on the basis of Archimedes’ law of buoyancy [7] with a resolution of 0.2 mm. In [8], a technique was developed using a wireless water-level transmitter that can measure the height of a fluid level in any container from a remote location using PC-based supervisory control with LabVIEW software-2023 Version, with an NI-6002 DAQ and an NI-USRP 2922. A capacitive coupled conductivity electrode is used to measure the liquid level in the narrow capillary for the range of 900 mm, with a resolution of approximately 0.2 mm, as reported in [9]. Reference [10] reported the design of cylindrical capacitance level sensors with varying cross sections for measuring the level of microdroplets for the angle range of 0–40%, with a sensitivity of 0.77 fF/µL. In [11], a capacitance liquid-level sensor was designed via analytic techniques with finite element analysis for improved sensitivity. 3D-printed capacitance sensors using nanotubes are designed to measure liquid levels within the range of 0–15 mm and are used for biomedical applications [12]. The background of the latest liquid-level sensing mechanisms using the capacitance principle is discussed to show the reported works for the design of electrodes and for improving sensor characteristics and performance. In addition, Table 1 summarizes the techniques used to increase sensitivity.

Generally, various techniques using different sensors have been used to increase sensitivity within a specified range. The sensitivity enhancement achieved via structural analysis and modifications of multiple sensors has been reported. The sensitivity of CLS was enhanced as follows:

(i) Selecting a suitable higher surface area of the electrodes;
(ii) Adding electrodes to the system within the operating range.

Several studies have reported diverse applications of capacitive sensors, which are discussed here. An integrated complementary metal oxide semiconductor capacitive sensor array provides a dynamic range of operations in life science applications, as detailed in [20]. The application of a capacitive grid sensor for the measurement of linear displacement is detailed in [21], with a maximum error of 1.5 mm within a specific displacement range. The detection of space gravitational waves via a six-degrees-of-freedom capacitance sensor with a stable calibration technique was reported in [22]. In [23], the design of a capacitance-level sensor electrode for fuel measurement in a helicopter fuel tank was reported, and designs such as the use of vertical slits and horizontal slits in electrode plates were compared. The design of a multiplexed parallel plate arrangement for measuring the liquid level via the capacitance change principle was reported in [24], and the results of the multiplexed parallel capacitance were compared with those of the conventional parallel plate arrangement. Neural network algorithms were used for temperature scaling adaptive measurements in [25]. This technique uses deep neural algorithms as classifiers to distinguish the scaling factors and obtain an automated relation of measurement variables.

In summary, the survey highlighted a primary focus on sensitivity enhancement within specific ranges, with little exploration of broader ranges or range tuning. Notably, there was no mention of autoranging for sensitivity improvement. This paper addresses this gap by proposing an autorange feature for a novel helical capacitance sensor to enhance sensitivity across any user-defined range. The sensor automatically adjusts the resolution within the standard 0–60 cm output range, ensuring consistent sensitivity. This work advances the field by improving helical CLS sensitivity via neural networks.

In this work, the performance characteristics of the capacitance level measurement system are distinguished according to their metrological meanings. The measurement range represents the interval of liquid levels over which the system is operated. The intrinsic sensitivity of the capacitive sensing element describes the change in the capacitance with respect to the liquid level, whereas the output sensitivity of the complete measurement chain describes the change in the conditioned output voltage with respect to the liquid level. Resolution represents the smallest change in the liquid level that can be reliably distinguished by the complete measurement system and therefore depends on the sensor response, electronic noise, ADC resolution, repeatability, hysteresis, drift, and environmental conditions. The accuracy describes the agreement between the measured liquid level and an independently established reference value. Hysteresis represents the difference between outputs obtained at the same liquid level during filling and draining cycles, whereas repeatability quantifies the variation among measurements obtained under identical conditions. These characteristics are treated separately in the following analysis to avoid interpreting an increase in output sensitivity as an automatic improvement in accuracy or physical resolution.

## 2. Process Analysis via Helical CLS

**Figure 1:**
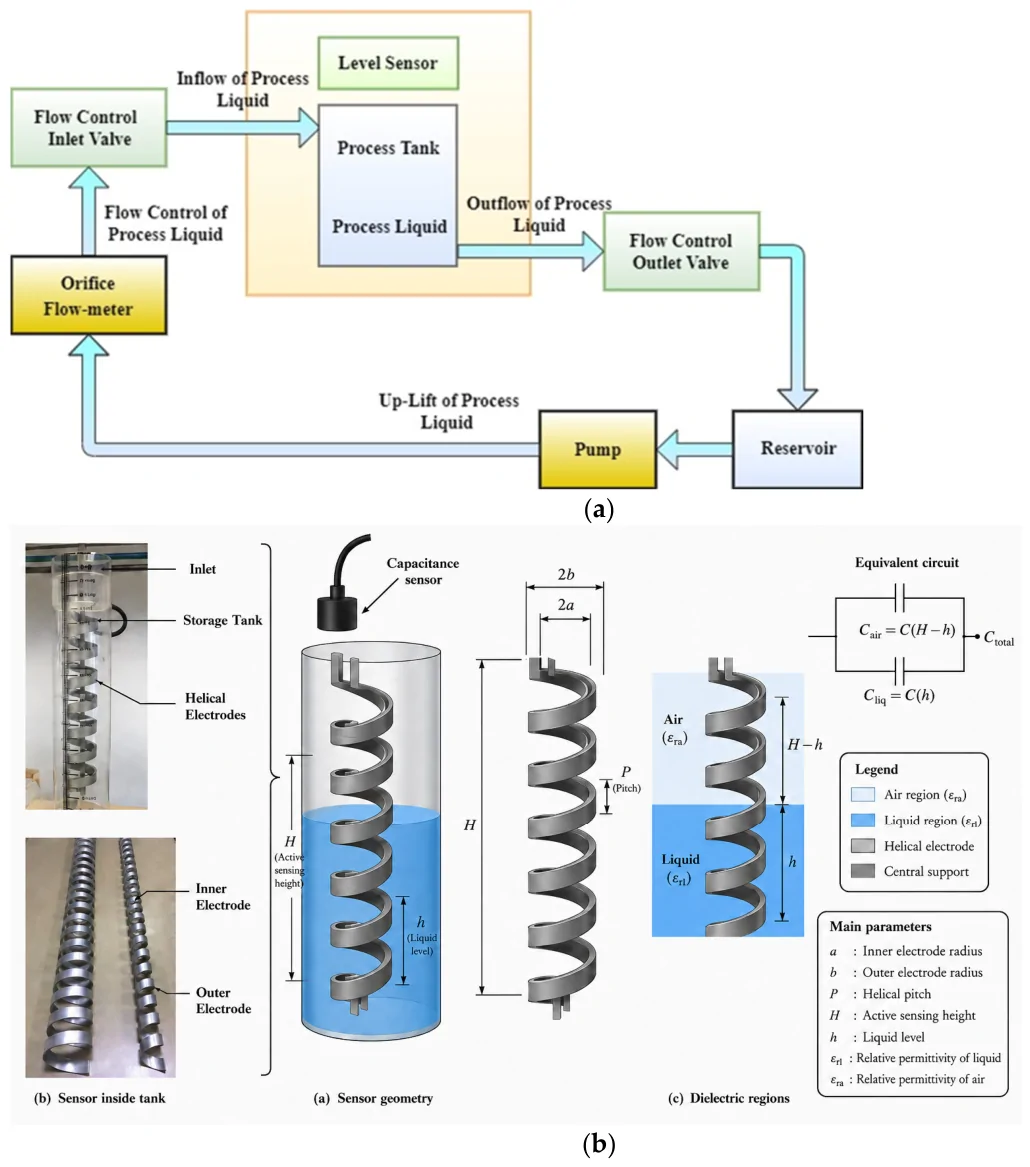
(**a**) Block diagram and (**b**) process diagram of a level process.

**Figure 2:**
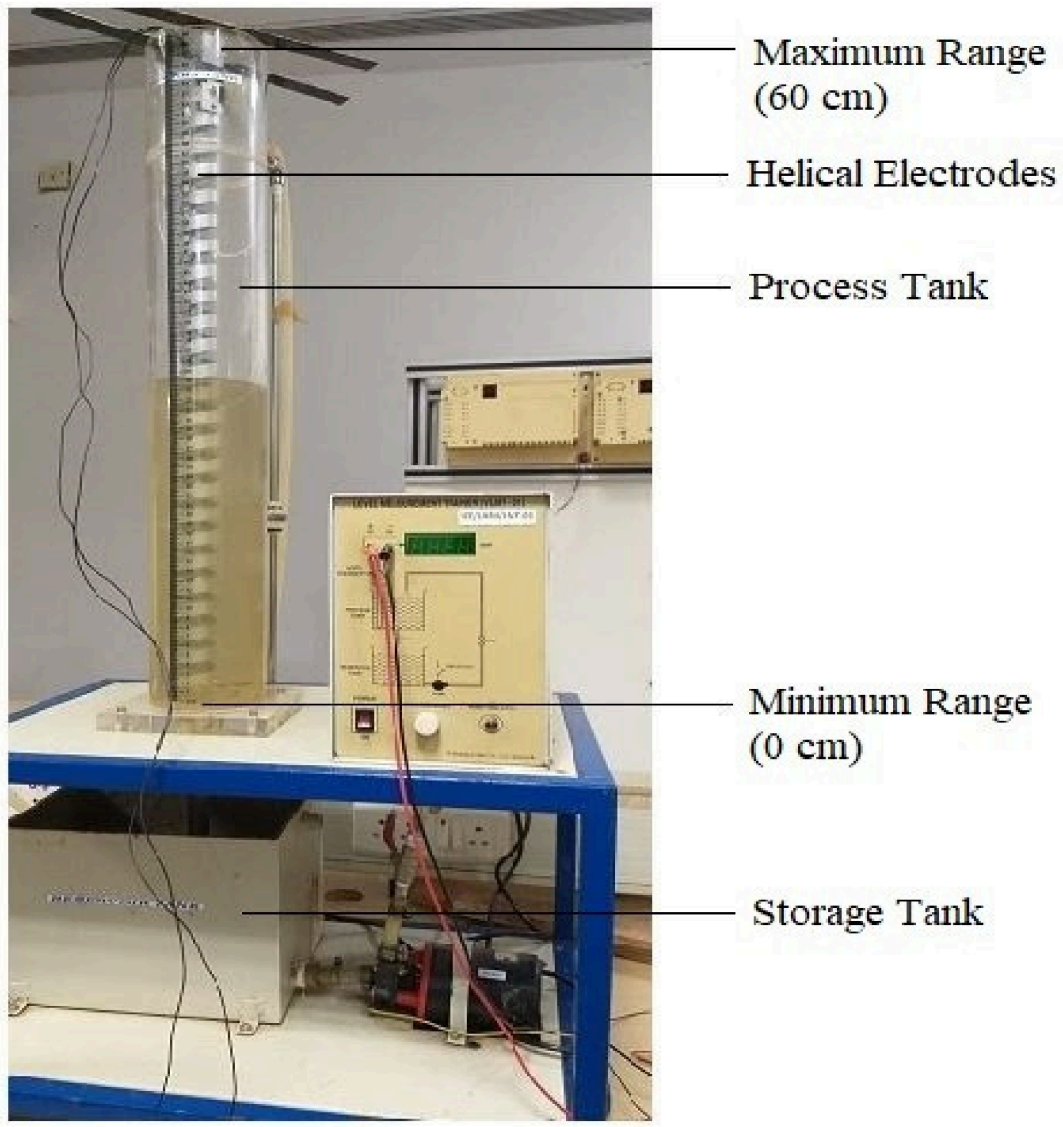
Experimental setup.

The helical CLS operates on the principle that the capacitance changes with the liquid level because of the variation in the dielectric medium between two electrodes [26,27]. It is constructed by placing two identical, novel helical-structured sensing electrodes into a process tank. A block diagram of a level process is shown in Figure 1. CLS forms capacitors parallel to the liquid and air in the tank. This research focuses on using the helical CLS to measure the level change of a single liquid at a time, as depicted in Figure 2. Compared with other CLSs, the novel design has greater sensitivity because of its structure, but the primary focus is to increase the sensitivity for different user-defined ranges; hence, a novel autorange technique is proposed. The capacitance output for a change in the liquid level is expressed by Equation (1).

(1) $$C_{Th}=\frac{2\pi \varepsilon_{o}\varepsilon_{ra}(H-h)}{\ln\left[\frac{b}{a}\right]+\ln\left[\frac{P+\sqrt{P^{2}+{\left(2\pi a\right)}^{2}}}{P+\sqrt{P^{2}+{\left(2\pi b\right)}^{2}}}\right]}+\frac{2\pi \varepsilon_{o}\varepsilon_{rl}(h)}{\ln\left[\frac{b}{a}\right]+\ln\left[\frac{P+\sqrt{P^{2}+{\left(2\pi a\right)}^{2}}}{P+\sqrt{P^{2}+{\left(2\pi b\right)}^{2}}}\right]}$$

where C_Th_ is the total helical capacitance value, H is the maximum height tank, h is the liquid level, P is the pitch, a is the outer electrode radius, b is the inner electrode radius, ε_o_ is the absolute permittivity, ε_rl_ is the relative permittivity of the liquid and ε_ra_ is the relative permittivity of air. The following subsection discusses the liquid level measurement, signal conversion, and signal condition circuit, which converts the output of the CLS, i.e., the capacitance, to a standard voltage.

### Output Conversion of CLS

The output of the helical CLS with respect to the capacitance was measured with respect to the change in the liquid level. The obtained capacitance was converted into an AC voltage via De Sauty’s bridge. The AC voltage obtained was in millivolts, which was conditioned to DC voltage with a range of 0–3 V by a signal-conditioning circuit.

**Figure 3:**
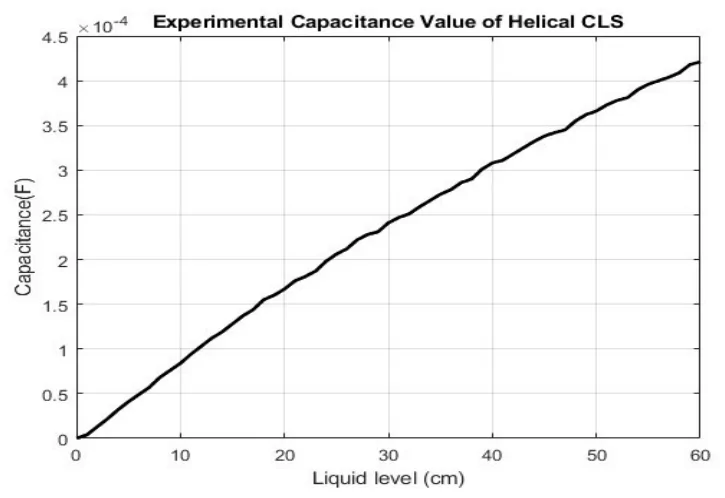
Experimental capacitance values of helical CLS.

*Capacitance Measurement*: The helical CLS was tested by immersing its helical electrodes in a 70 cm nonconductive tank. When a dielectric liquid was introduced, it acted as the medium between the electrodes, causing a change in capacitance that corresponded to the liquid level. The calibrations included empty and full tank measurements. The capacitance was measured at 1 cm intervals as the fluid level increased to 60 cm. The highest capacitance for the 60 cm level was 421 µF, as shown in Figure 3.

**Figure 4:**
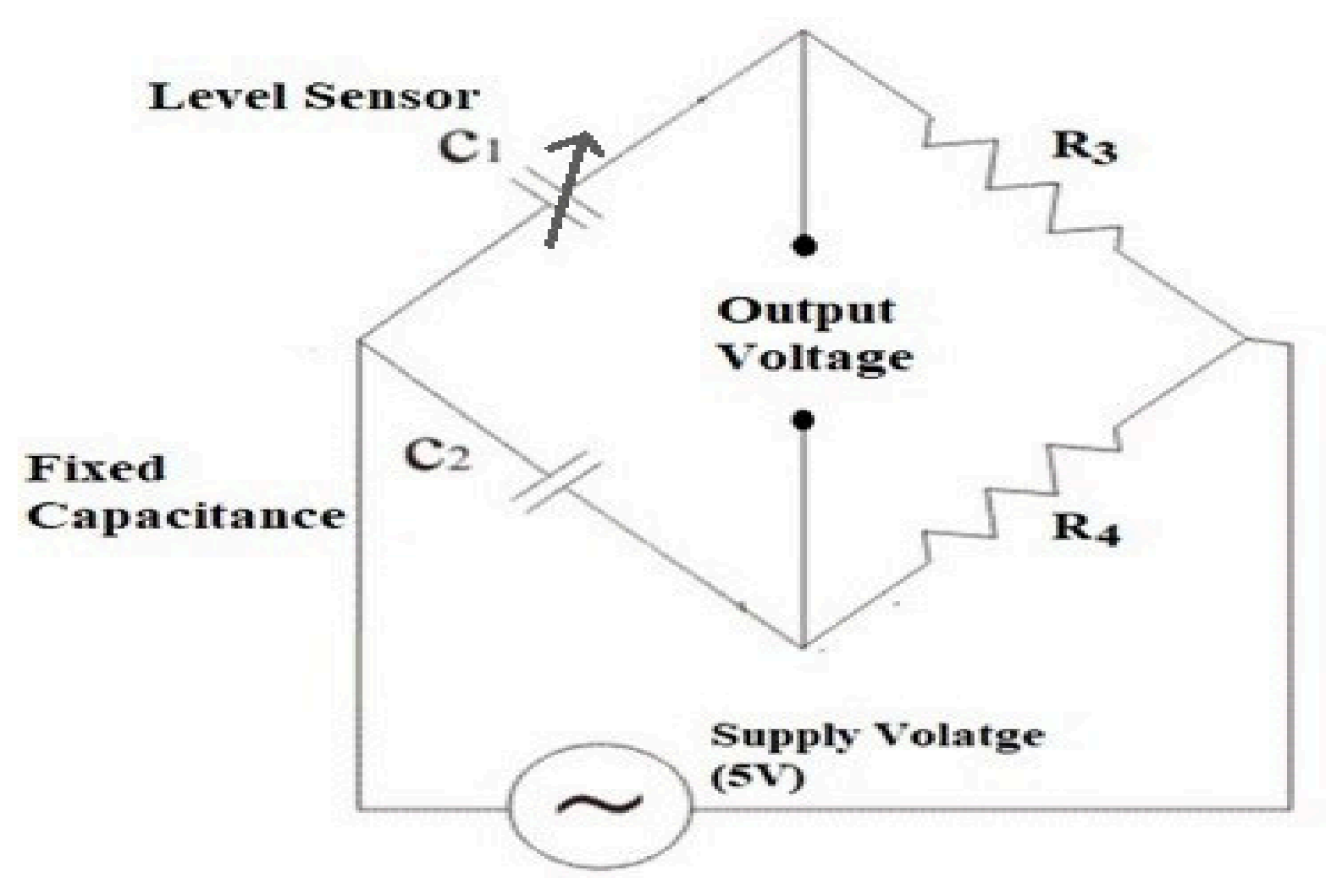
Capacitance measurement via De Sauty’s bridge.

*Capacitance-to-voltage conversion*: The liquid level is measured in terms of capacitance via CLS. The unknown capacitance is converted into a known voltage via De Sauty’s bridge. The bridge is designed by using two known resistances (R1 and R4), one known capacitance (C2), and one unknown capacitance (C1), which is the sensor output. The bridge for measuring the capacitance in terms of the voltage is shown in Figure 4.

**Figure 5:**
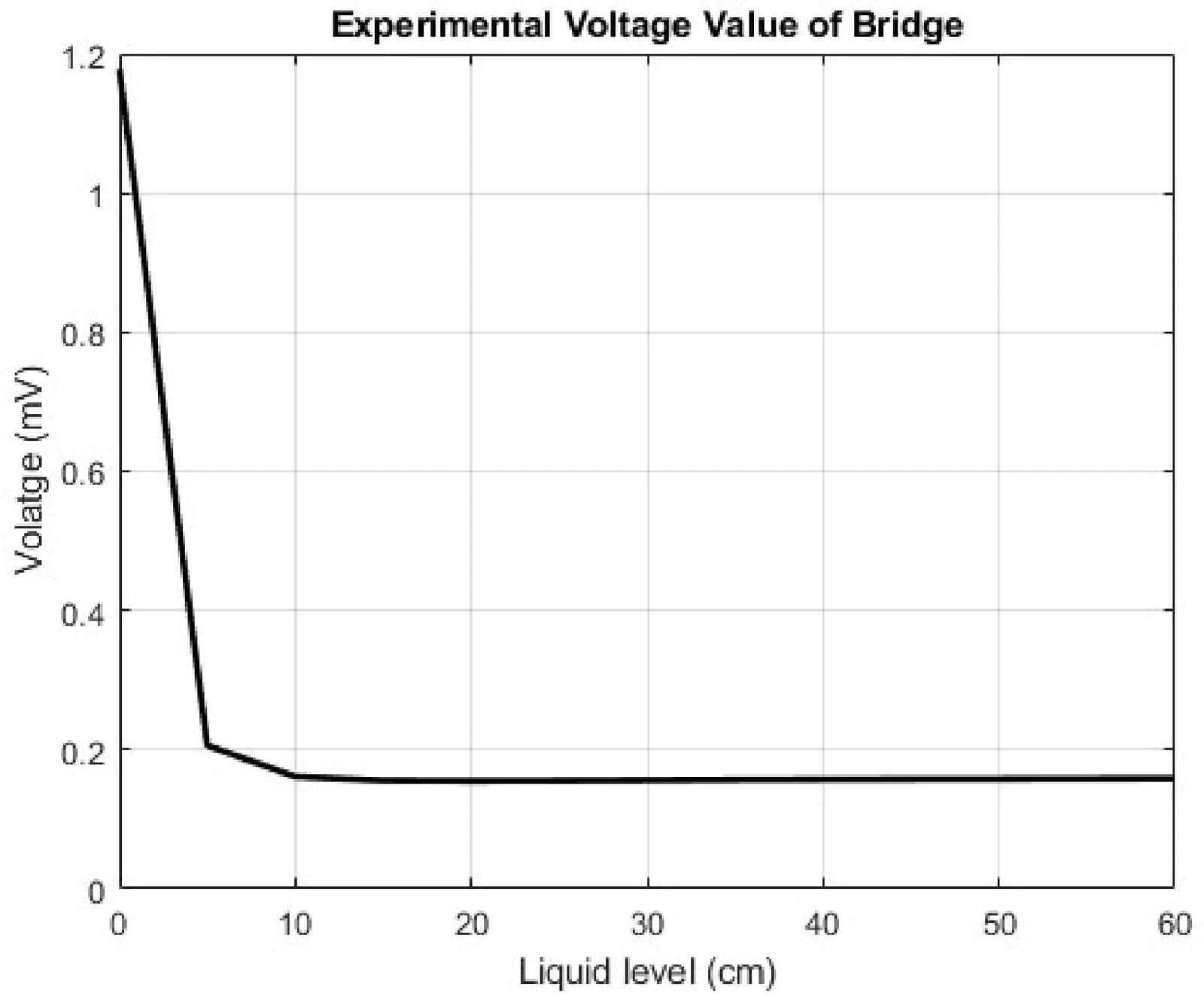
Bridge output voltage of the helical CLS.

The experiments were conducted according to the above specifications, and the results are shown in Figure 5. At 60 cm, the capacitance was 421 µF; for this level, the bridge output obtained was 0.158 mV.

**Figure 6:**
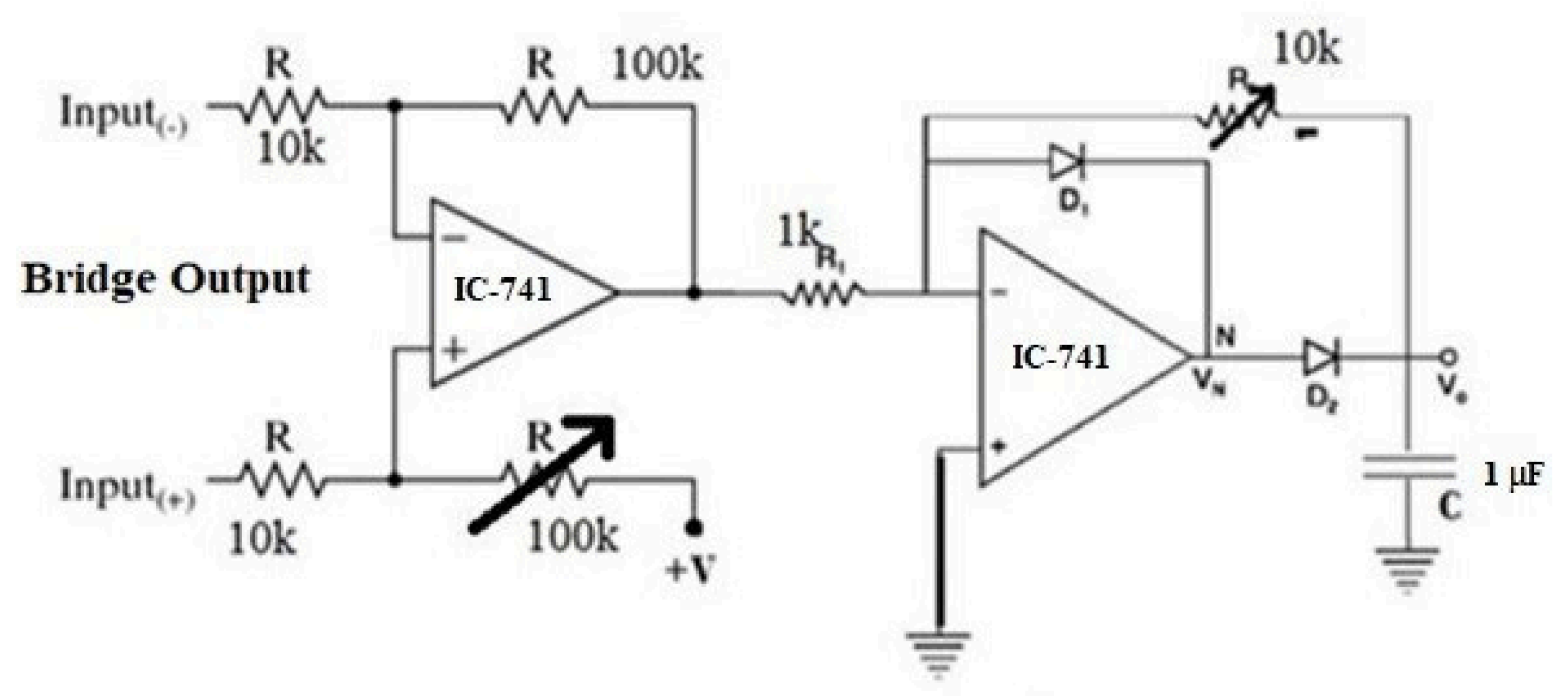
Signal conditioning circuit for helical CLS.

**Figure 7:**
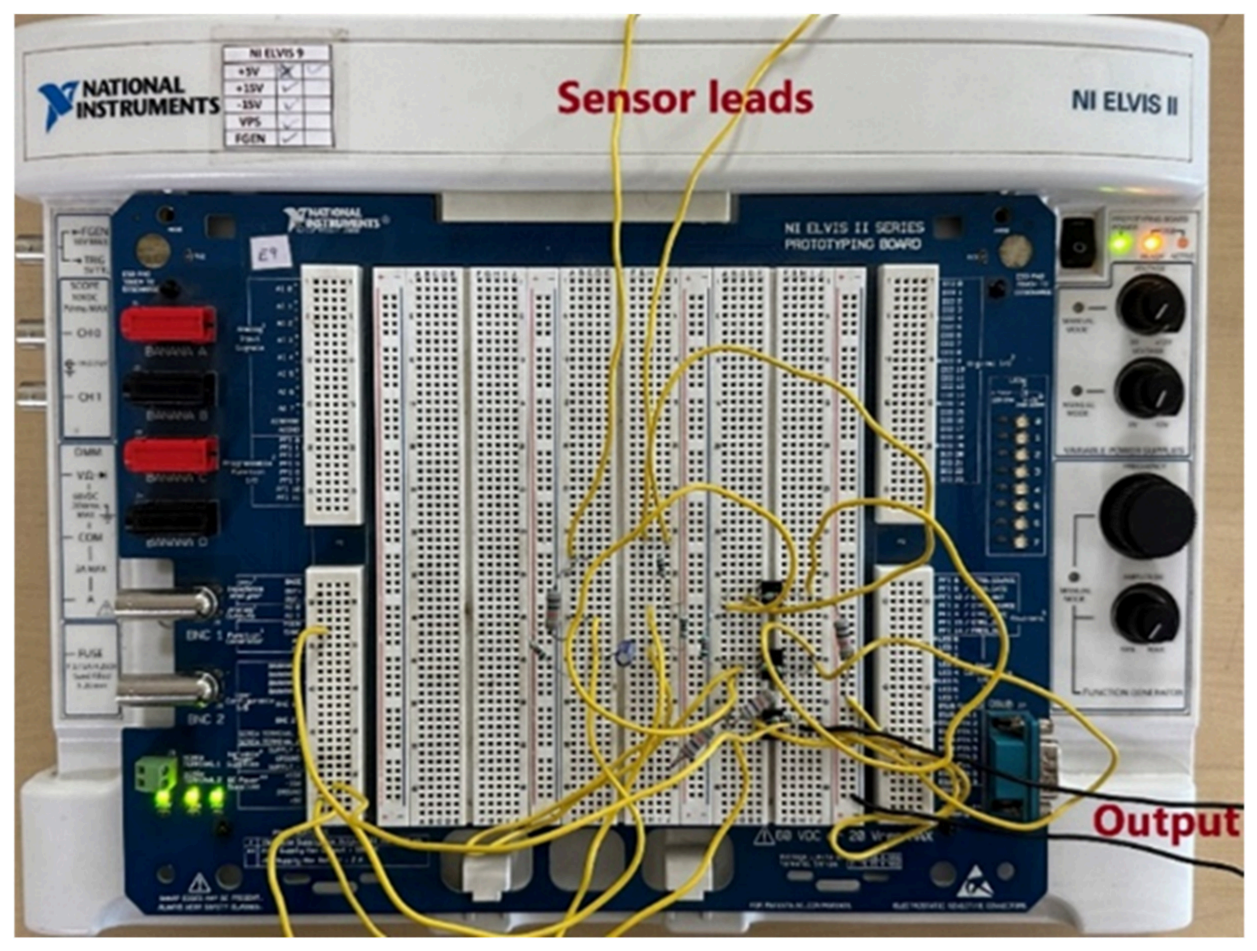
Practical configuration of a signal-conditioning circuit for helical CLS.

**Figure 8:**
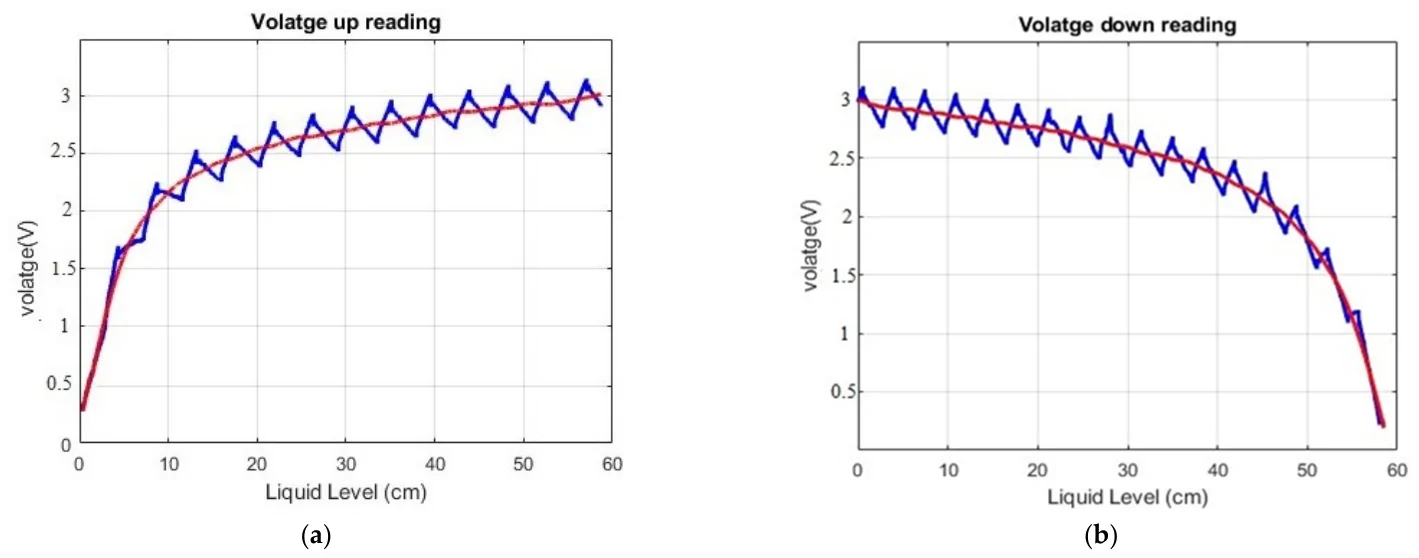
(**a**) Upper and (**b**) lower reading outputs of the signal-conditioning circuit for helical CLS.

*Signal conditioning circuit*: Signal conditioning is categorized into three stages: filtering, amplifying, and data acquisition. The circuit included resistors, an IC-741 amplifier, a DC power supply, diodes, capacitors, and an NI Elvis board. As shown in Figure 6 and Figure 7, the 100k potentiometer was set to display 1 V at a 5 cm liquid level. In contrast, the 10k potentiometer was adjusted to 3 V at the maximum level. Data were acquired via DAC cards on a PC, with readings taken during filling and draining. The output was smoothed with a low-pass filter, as illustrated in Figure 8 and detailed in Table 2.

**Figure 9:**
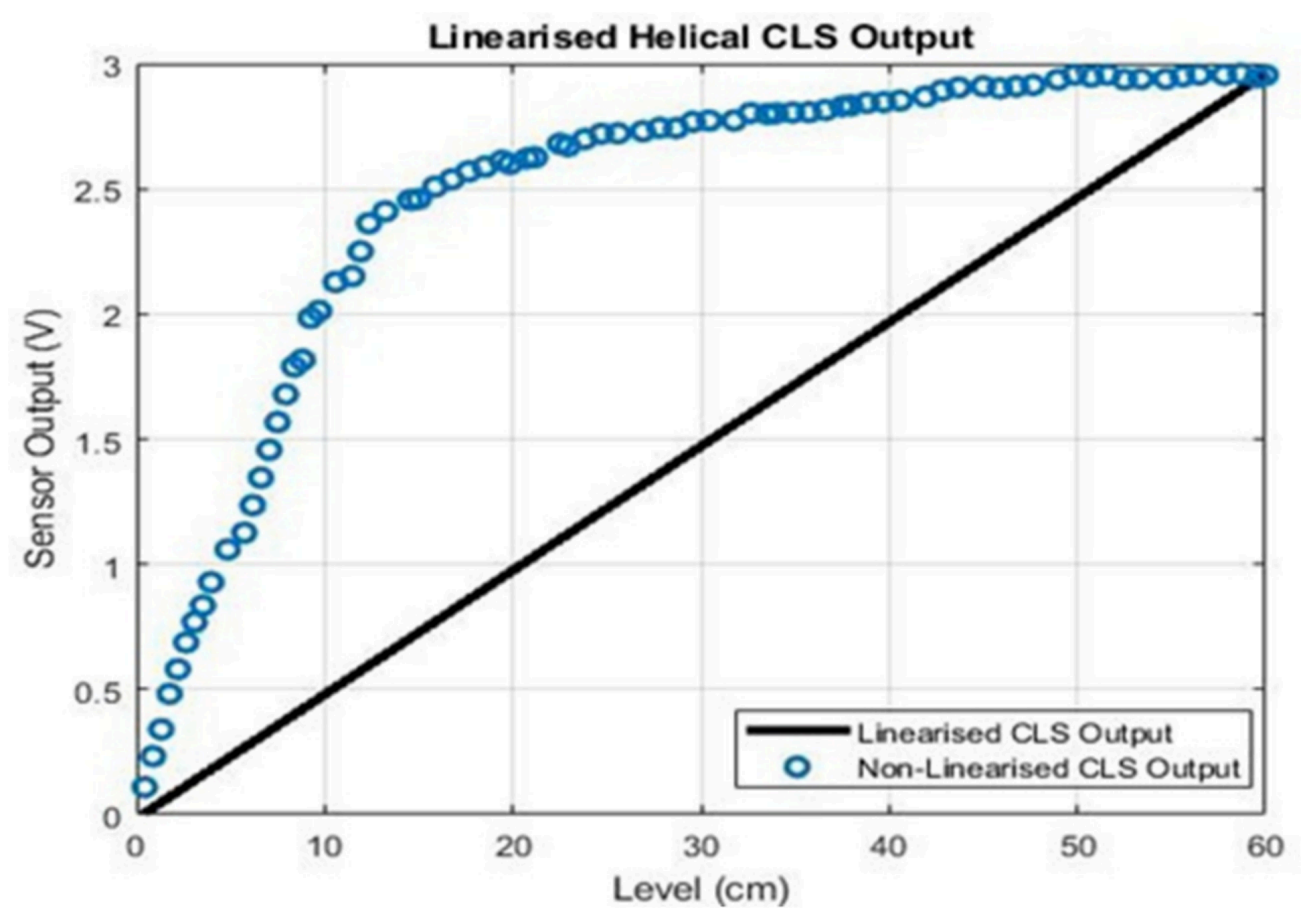
Linearized output of helical CLS.

*Output linearization*: The output is linearized via linear regression analysis [28]. The output voltage is linearized to the third-order degree equation, as given in Equation (2), where y is the output voltage, and x is the cm level. The linearized output is shown in Figure 9.

(2) $$y=2.269\times 10^{-5}(x^{3}-0.0036x^{2}+0.2035x+0.01914)$$

Equation (2) represents the conditioned sensor output voltage in volts and represents the liquid level in centimetres. Accordingly, the polynomial coefficients are dimensional quantities rather than dimensionless constants. For a third-order calibration relation, the corresponding coefficient units are V/cm^3^ for, V/cm^2^ for, V/cm for, and V for. These units have been explicitly stated to maintain the dimensional consistency of the calibration equation.

Compared with other sensors, the novel helical design has the highest sensitivity, but this work focuses on increasing the sensitivity for different user-defined ranges. Hence, linearization is given the greatest prominence to develop an autorange functionality by training the network.

All the static characteristics of the sensor with respect to the change in the liquid level are listed below.

The characteristics of the helical CLS are listed in Table 3. Additionally, the full-range sensitivity of the helical CLS is calculated as the ratio of the difference between the maximum and minimum output voltages (Vmax and Vmin) to the difference between the maximum and minimum level changes (Lmax = 60 cm, and Lmin = 0 cm), which is expressed in Equation (3).

(3) $$Full\;range\;sensitivity=\frac{V_{max}-V_{min}\;}{L_{max}-L_{min}}$$

Two different sensitivity definitions should be distinguished for the proposed system. The intrinsic capacitance sensitivity of the sensing element is defined locally as follows:

(4) $$S_{c}\left(h\right)=\frac{dC(h)}{dh}$$

where is the sensor capacitance and is the liquid level. In contrast, the effective output sensitivity of the complete conditioned measurement system is defined as follows:

(5) $$S_{v}\left(h\right)=\frac{dV_{0}(h)}{dh}$$

where V_{0} denotes the conditioned output voltage. For a finite operating interval, the corresponding average output sensitivity can be expressed as follows:

(6) $$S_{v,\;avg}=\frac{\Delta V_{0}}{\Delta h}$$

The proposed autoranging method does not alter the geometry of the helical electrodes nor the underlying capacitance–level relationship and therefore, should not be interpreted as increasing the intrinsic physical sensitivity of the sensing element. Instead, the effective output sensitivity is increased by mapping the electrical response associated with a selected liquid-level interval onto the available standardized output-voltage span. Accordingly, the sensitivity enhancement reported hereafter refers specifically to the effective output sensitivity of the complete measurement system.

An analysis was carried out to increase the sensitivity of the helical CLS for the obtained linearized output, and the problem statement is discussed in the section below.

## 3. Problem Statement

The sensitivity of the CLS is improved for specific operating ranges, but it maintains fixed sensitivity and resolution across different ranges. These limitations fail to effectively detect small changes in level when the range is adjusted. To overcome this, tests were conducted on a novel helical CLS to evaluate how changing the range affects sensitivity. This research focuses on using helical CLS for liquid-level measurements within a 0–60 cm range.

**Figure 10:**
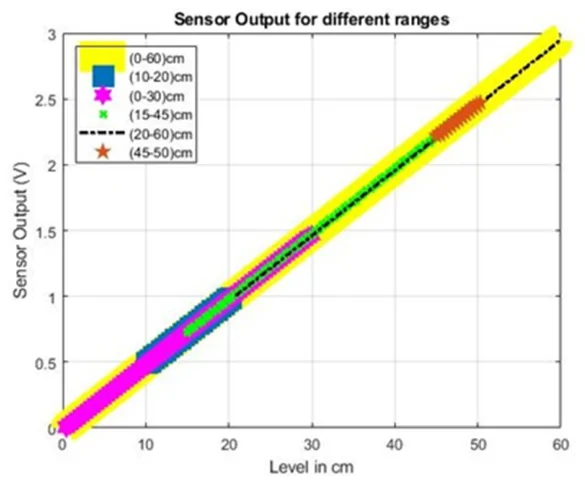
Helical CLS outputs for different ranges.

The sensor range is defined by its maximum and minimum measurement levels. The helical CLS yields 0.010939 V at 0 cm and 2.953 V at 60 cm, indicating high sensitivity and accuracy within this range. However, if the user selects a different range, the sensor struggles to detect small-level changes. Thus, a design that will automatically adjust the resolution and sensitivity on the basis of the user-defined range is needed. To evaluate how range changes affect sensitivity, six different operating ranges were tested: 0–60 cm, 10–20 cm, 0–30 cm, 15–45 cm, 20–60 cm, and 45–50 cm, as detailed in Table 4. The outputs for these ranges are illustrated in Figure 10.

From the input–output characteristics, the full-range sensitivity of the helical CLS is 4.904 V/m. The sensitivities for all the user-defined ranges are listed in Table 4. The graph shows that for the different user-defined ranges, the sensor’s minimum and maximum voltage outputs are unlike the standard output, i.e., (0.010939–2.953) V. The effect of the change in range on the sensitivity of the sensor is mentioned below.

- The helical CLS measures full-scale sensitivity for an operating range of 0–60 cm;
- These changes affect sensor resolution;
- The same sensor fails to measure the same sensitivity for different ranges selected by the user, as shown in Cases 1 to 6.

The sensor sensitivity varies with different user-defined ranges. The sensor’s minimum and maximum outputs must be adjusted according to the user-defined range to standardize the output voltage between 0.010939 V and 2.953 V and improve sensitivity across any range. A smaller input range typically allows for higher sensitivity. This research introduces an auto ranging functionality to address this issue, with detailed development in the following section.

## 4. Problem Solution

**Figure 11:**
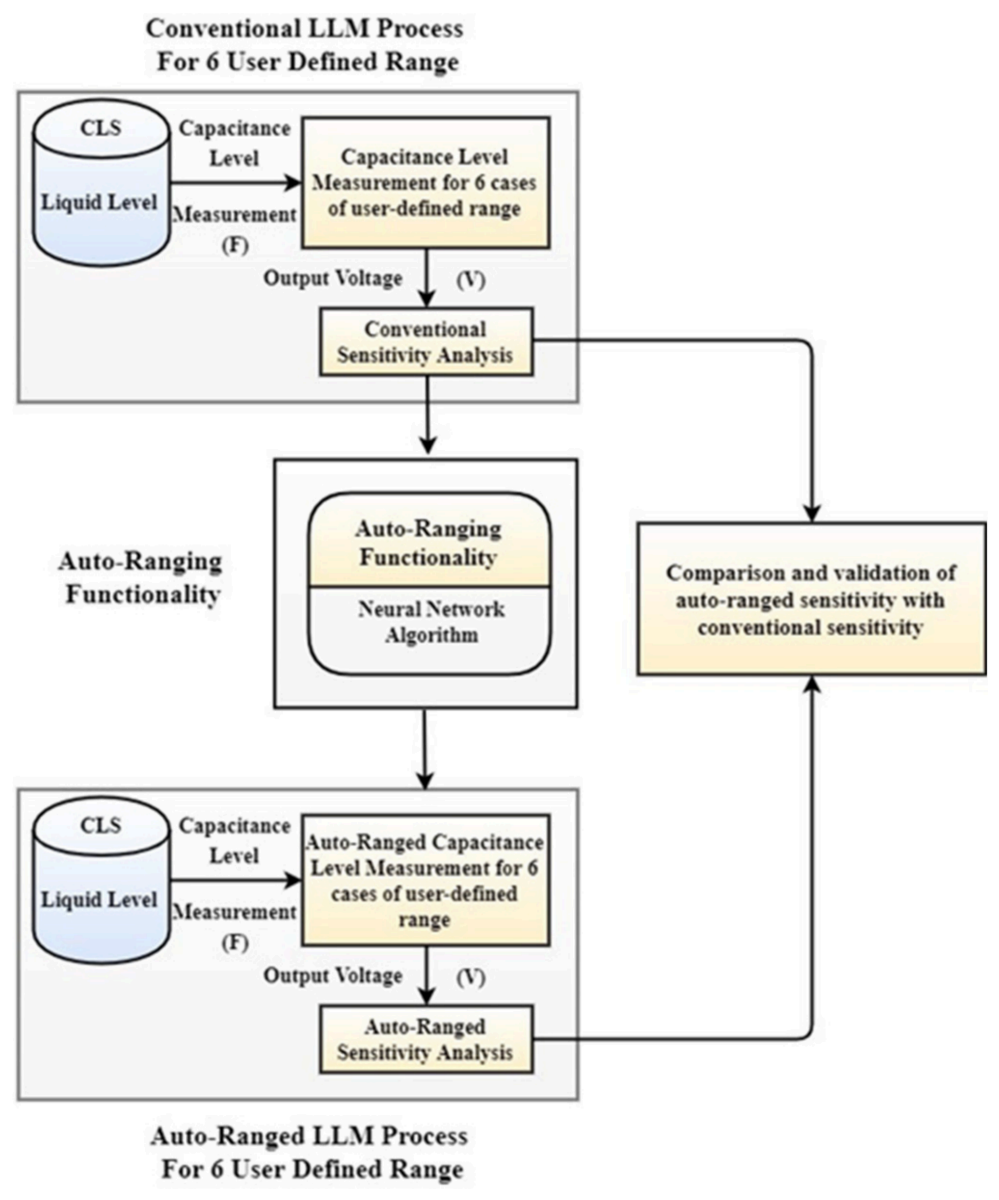
Process analysis for the design and development of autorange functionality.

The main objective of this paper is to increase the sensitivity of the helical CLS for any user-defined range. In this paper, a novel autorange technique is proposed to overcome this disadvantage. This functionality enhances the sensor’s sensitivity for any user-defined range, which is selected between the operating ranges of the sensor. The autorange functionality allows the sensor to read even a small change in level for any user-defined range, and the development process of the autorange functionality is shown in Figure 11 below.

The helical CLS is used for liquid-level measurements with a fixed operating range of 0–60 cm. Here, the range of the sensor is defined by the maximum and minimum heights of the sensor. The sensor gives an output of 0.010939 V for the 0 cm level and 2.953 V for the maximum range, i.e., for the 60 cm level. The resolution is mapped as 0.010939 V for 0 cm and 2.953 V for 60 cm, which is stated to be 0.1 cm, and it accurately measures the liquid level with high sensitivity within this range. The operating specifications are given in Table 5.

Furthermore, to develop an autorange functionality for an LLM system, six different user-defined ranges are selected for the purpose of design, development and implementation of the functionality, i.e., Case 1, Case 2, Case 3, Case 4, Case 5, and Case 6 for ranges of (0–60) cm, (10–20) cm, (0–30) cm, (15–45) cm, (20–60) cm and (45–50) cm, respectively. This paper discusses the development of an autorange technique using a neural network model. The neural network algorithm is trained to map the input range defined by the user to the standard signal of 0.010939 to 2.953 V via the feed-forward back propagation network. The development of an algorithm is discussed in the following subsection.

### Development of the Neural Network Model

An autorange model was developed using a neural network (NN). This network adjusts its parameters by comparing the network output and the target output until they match. The NN model maps the output of the helical CLS for various user-defined ranges to a standard voltage range of 0.010939–2.953 V. The inputs to the network include sensor outputs for different ranges, whereas the targets are voltages within the 0.010939–2.953 V range. Training the network aims to produce the desired output from given inputs. Table 6 details the model specifications, which include a trained NN with two hidden layers and 10 neurons.

For an ideal linear measurement characteristic, autoranging between an input interval [V_L_, V_H_] and a desired output interval [V_OL_, V_OH_] can be achieved using the affine transformation

(7) $$V_{o}=V_{OL}+\frac{\left(V-V_{L}\right)}{{(V}_{H}-V_{L})}(V_{OH}-V_{OL})$$

Therefore, the use of an ANN is not required solely for linear range scaling. In the present system, however, the measured response includes nonlinearities introduced by the capacitance–level characteristics, bridge conversion, signal-conditioning electronics, and experimental variations. The ANN is consequently employed as a data-driven nonlinear calibration function that learns the input–output relationship without requiring an explicit analytical inverse model of the complete measurement chain. Conventional approaches such as polynomial calibration, lookup tables, and piecewise interpolation can also be used for this purpose; the ANN provides an alternative framework capable of representing nonlinear mappings and being extended to additional operating variables in future implementations.

**Figure 12:**
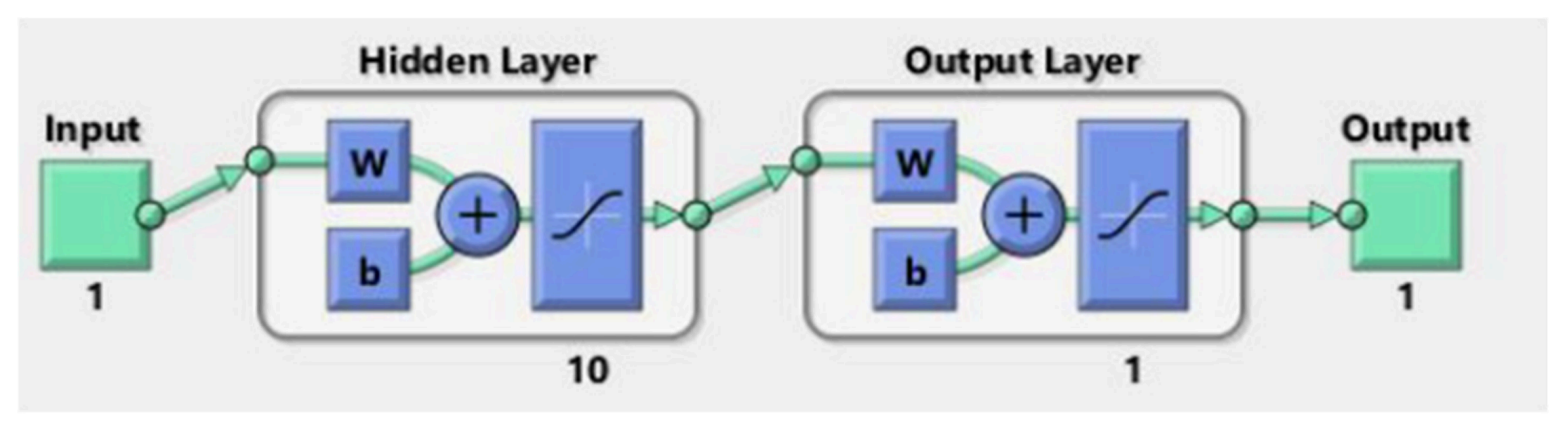
Schematic of the neural network model.

A feedforward backpropagation model, the Levenberg–Marquardt training function, and the mean square error (MSE) performance function are used to train the neural network. It consists of hidden layers, neurons, weights, and neuron transfer functions, which are varied to obtain the desired mean square error and regression. A mean square error of 0 and a regression value of 1 are always desired. In the proposed work for the constraints subjected, a neural network model is obtained, with two hidden layers having 10 neurons in each of the hidden layers. The tansig transfer function is used, as shown in Figure 12.

Data partitioning and model validation: For each of the six user-defined operating ranges, the available input–target data were randomly divided into three subsets, with 70% of the samples used for training, 15% for validation, and 15% for testing. The training dataset was used to optimize the weights and biases of the feed-forward neural network using the Levenberg–Marquardt backpropagation algorithm. The validation dataset was used to monitor the generalization capability of the network during training and to prevent overfitting, whereas the testing dataset was kept independent of the parameter update process and was used to evaluate the performance of the trained network on unseen samples. The same data-partitioning strategy was employed for all six operating-range cases to maintain consistency in the model evaluation. Model performance was assessed using the mean square error (MSE) and regression coefficient (R), with a low MSE and an R value close to unity, indicating good agreement between the network predictions and the target outputs.

**Figure 13:**
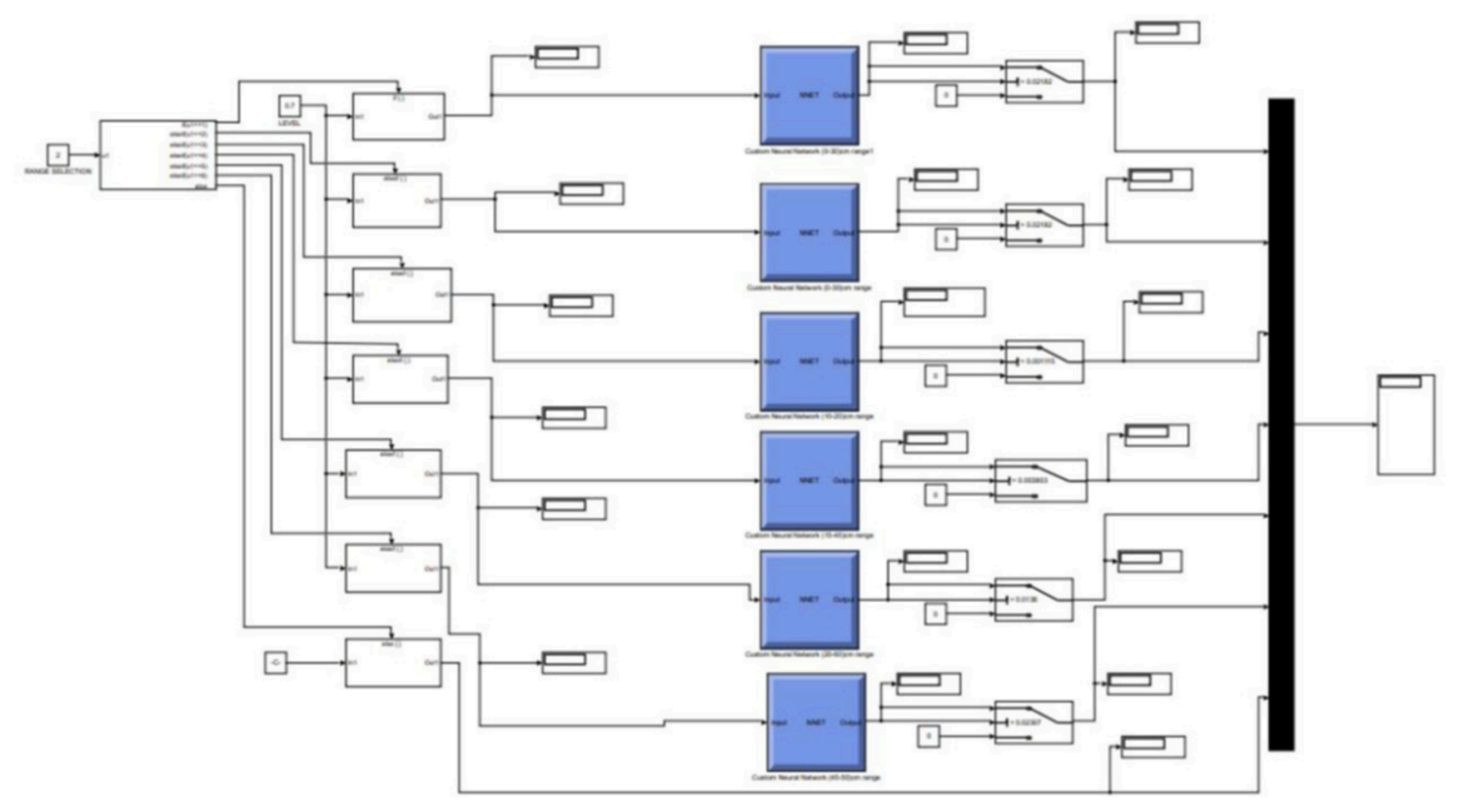
Combined neural network for all the different range functions.

All the trained neural networks are combined into a model via MUX. All the cases are incorporated into the model by defining Cases 1, 2, 3, 4, 5 and 6 as 1, 2, 3, 4, 5, and 6, respectively. Users can select any defined range as an input to the model, which results in the output being in the standard voltage range. The combined neural network model, which includes all the trained networks, is shown in Figure 13.

Notably, the present implementation demonstrates auto ranging for six predefined user-selectable operating ranges: 0–60 cm, 10–20 cm, 0–30 cm, 15–45 cm, 20–60 cm, and 45–50 cm. Separate trained mappings corresponding to these ranges are combined using a multiplexer-based selection mechanism. Therefore, the present model should not be interpreted as automatically generating a new calibration for an arbitrary pair of previously unseen values. Generalization to continuously selectable measurement limits would require either a unified model in which the lower and upper range limits are included as network inputs or an adaptive online calibration procedure. This constitutes an important direction for future development.

Physics-guided interpretation of the autoranging model: The operation of the proposed neural network-based autoranging model can be interpreted from the physical characteristics of the helical capacitance level sensor. An increase in the liquid level increases the portion of the helical sensing electrodes surrounded by the liquid dielectric, resulting in a corresponding variation in the effective capacitance of the sensor. This capacitance variation is converted into an electrical voltage through the De Sauty bridge and subsequent signal-conditioning stages. Thus, the electrical input supplied to the neural network inherently represents the physical relationships among the liquid level, dielectric interaction, sensor capacitance, and conditioned output voltage. When only a restricted portion of the complete 0–60 cm measurement range is selected, the conventional sensor utilizes only a fraction of its available output-voltage span, thereby limiting the voltage variation per unit change in the liquid level. The proposed neural network model learns the nonlinear transformation required to map this restricted sensor response onto the standardized full-scale output range of 0.010939–2.953 V. Consequently, sensitivity enhancement is achieved through intelligent autoranging of the measured sensor response rather than through modification of the physical sensing electrodes.

## 5. Results and Analysis

With all the training specifications, different cases are trained to obtain outputs within the specified standard range of 0.010939 to 2.953 V. The minimal MSE is obtained, with an R value of 0.999 in all the cases. All the considerations for training the network in different cases are discussed below.

**Figure 14:**
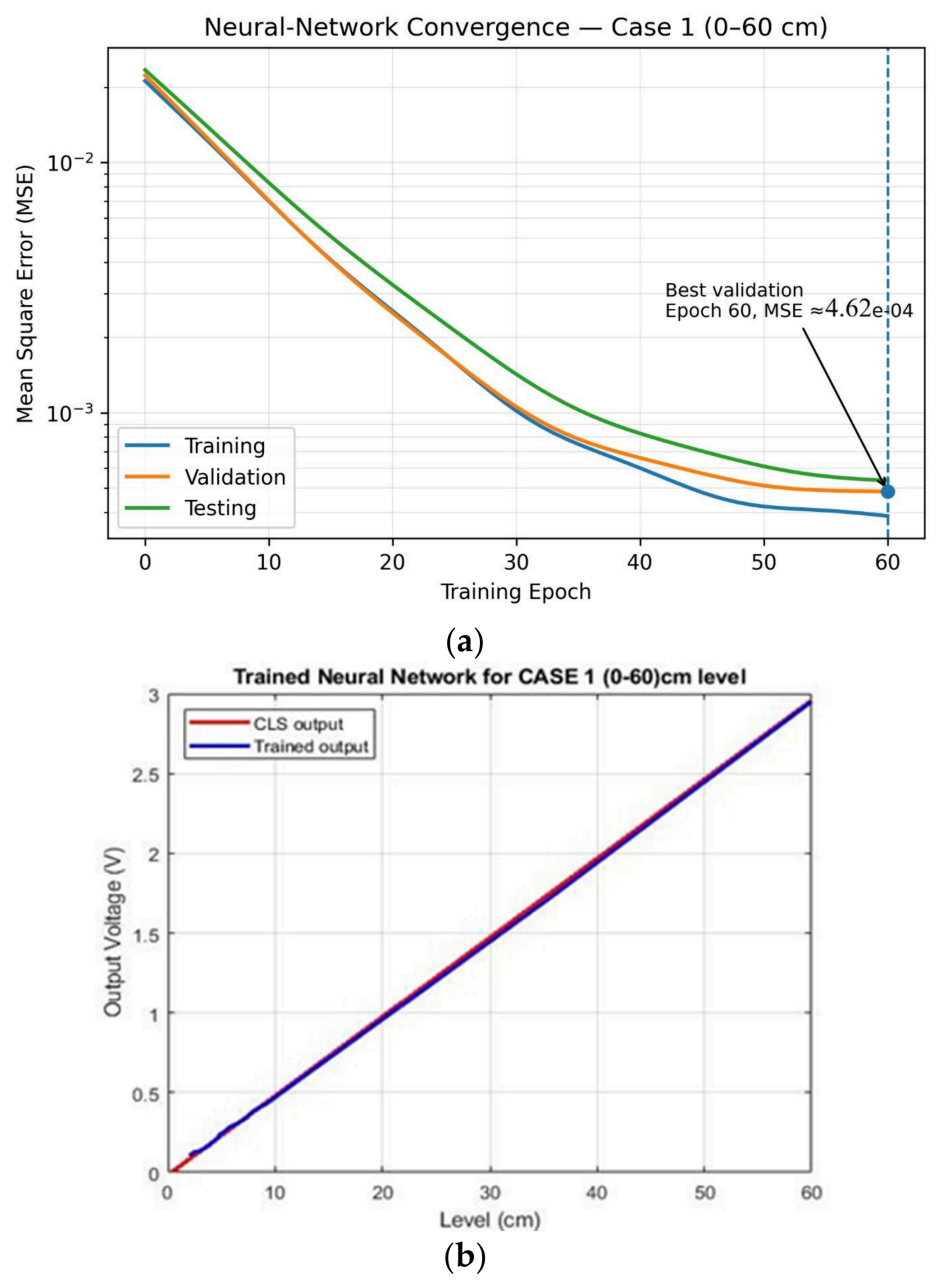
(**a**) Convergences curve of NN. (**b**) Case 1: Output of trained and untrained networks.

Case 1: Works for a 0–60 cm set range. The network is trained to obtain an R value of 0.9972 with an MSE less than 0.000462. The convergence curves of the NN and outputs of the trained network and untrained sensor are shown in Figure 14.

**Figure 15:**
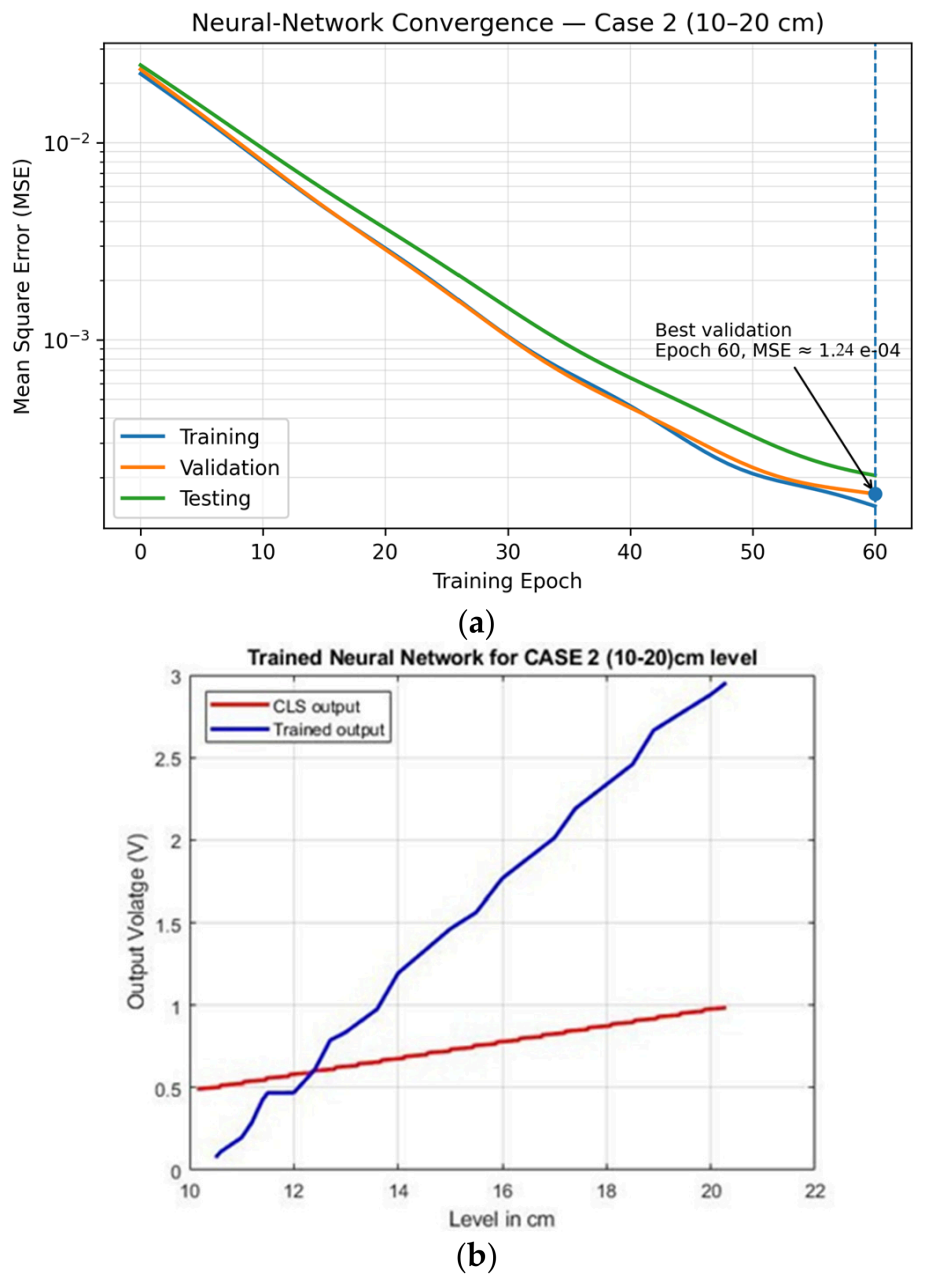
(**a**) Convergences curve of NN. (**b**) Case 2: Output of trained and untrained networks.

Case 2: Works for a set range of 10–20 cm. The network is trained to obtain an R value of 0.99872 with an MSE less than 0.000124. The convergence curves of the NN and outputs of the trained network and untrained sensor are shown in Figure 15.

**Figure 16:**
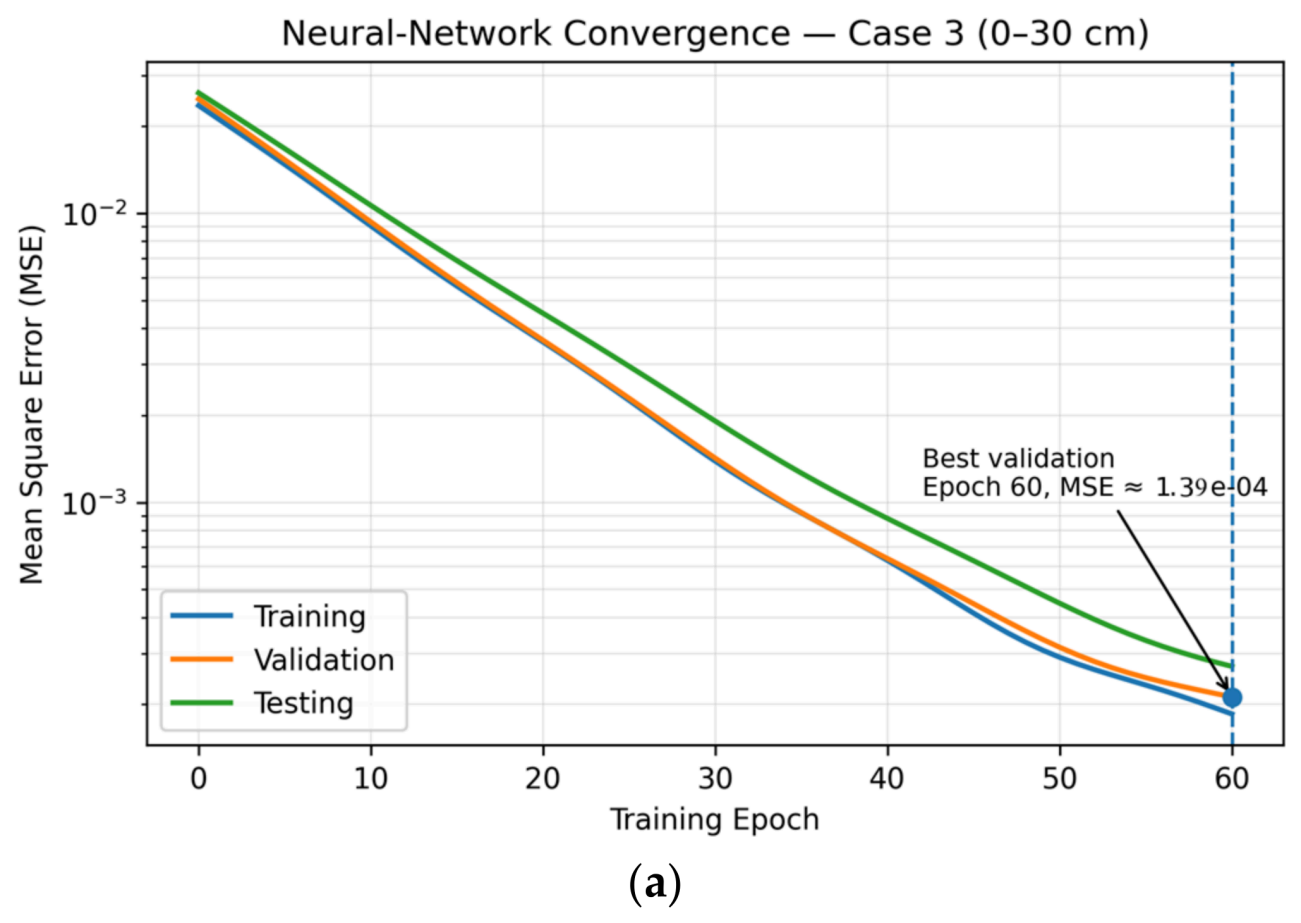

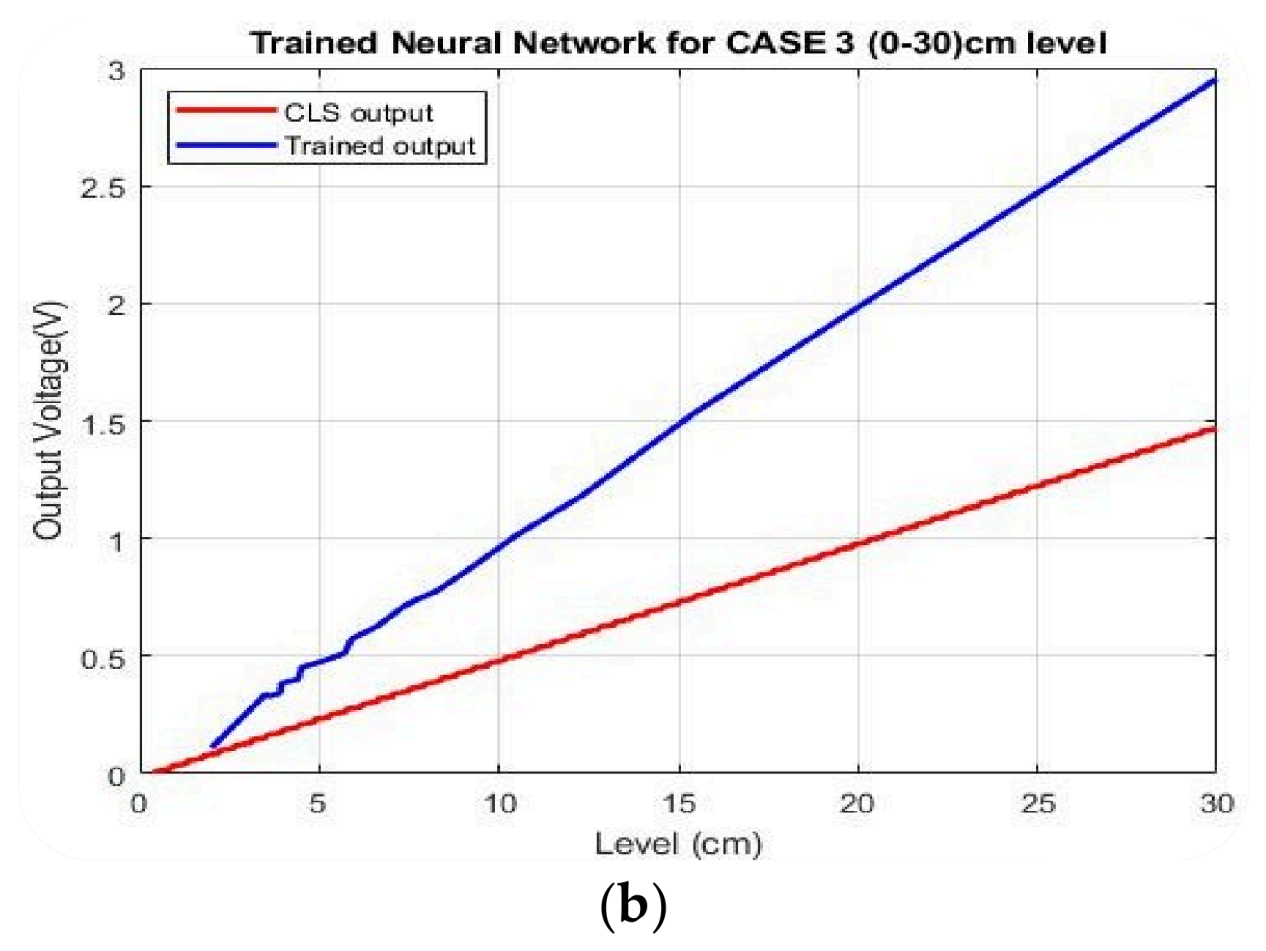
(**a**) Convergences curve of NN. (**b**) Case 3: Output of trained and untrained networks.

Case 3: Works for a set range of 0–30 cm. The network is trained to obtain an R value of 0.99882 with an MSE less than 0.000139. The convergence curves of the NN and outputs of the trained network and untrained sensor are shown in Figure 16.

**Figure 17:**
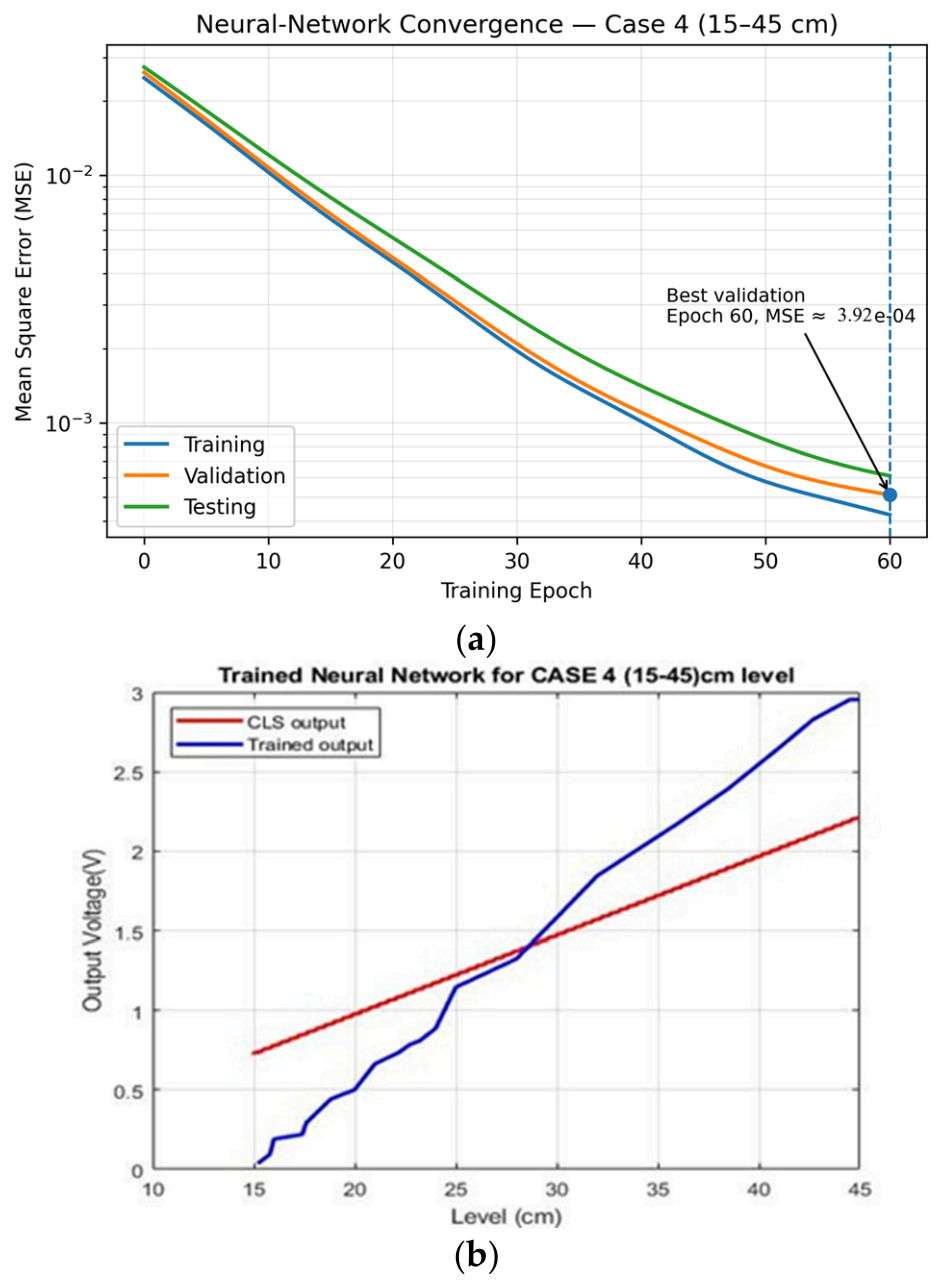
(**a**) Convergences curve of NN. (**b**) Case 4: Output of trained and untrained networks.

Case 4: Works for a set range of 15–45 cm. The network is trained to obtain an R value of 0.99752 with an MSE less than 0.000392. The convergence curves of the NN and outputs of the trained network and untrained sensor are shown in Figure 17.

**Figure 18:**
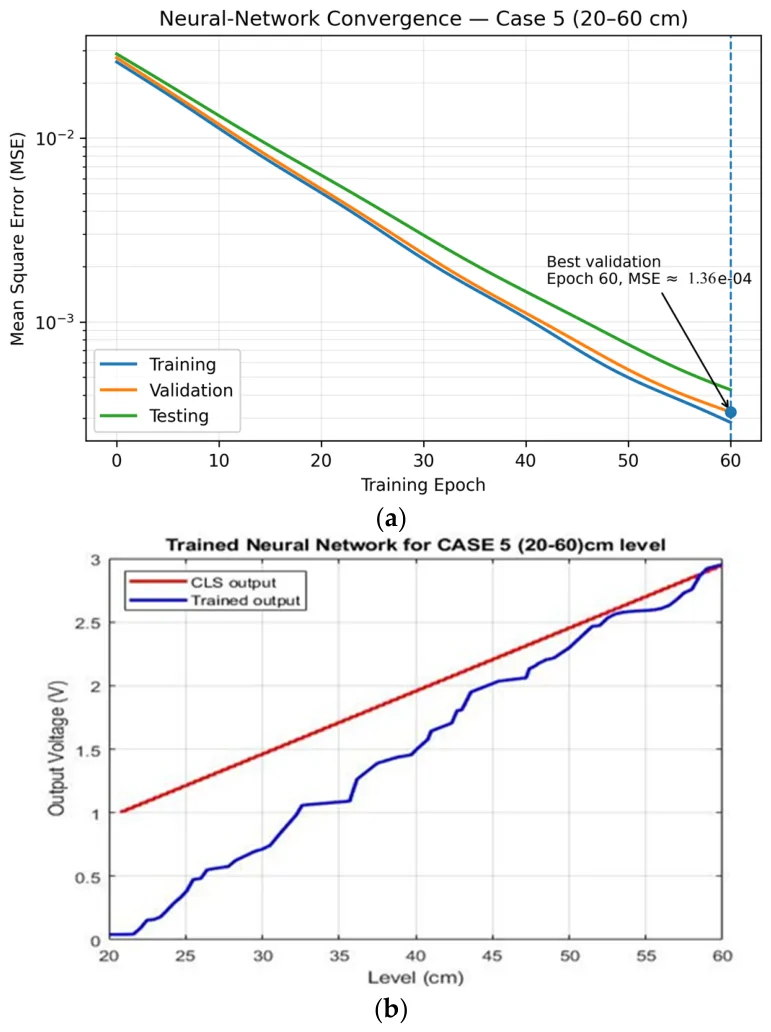
(**a**) Convergences curve of NN. (**b**) Case 5: Output of trained and untrained networks.

Case 5: Works for a set range of 20–60 cm. The network is trained to obtain an R value of 0.99321 with an MSE less than 0.000136. The convergence curves of the NN and outputs of the trained network and untrained sensor are shown in Figure 18.

**Figure 19:**
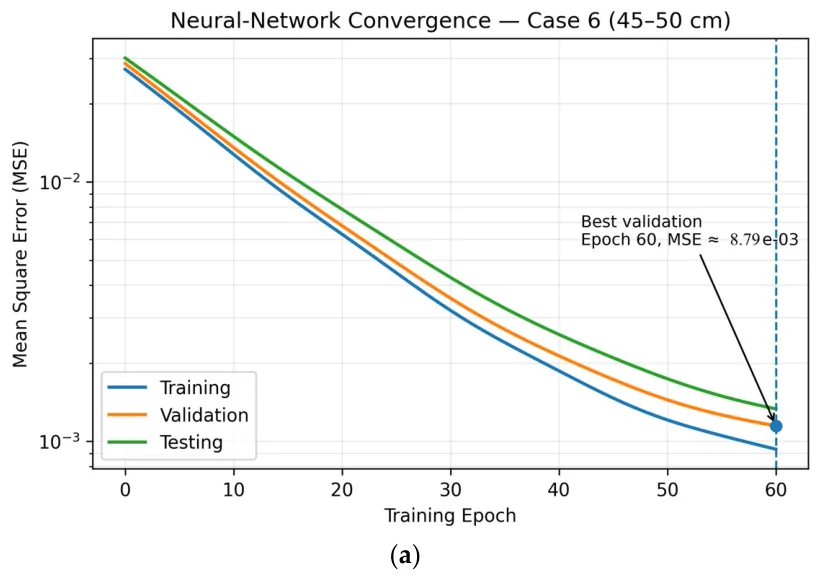

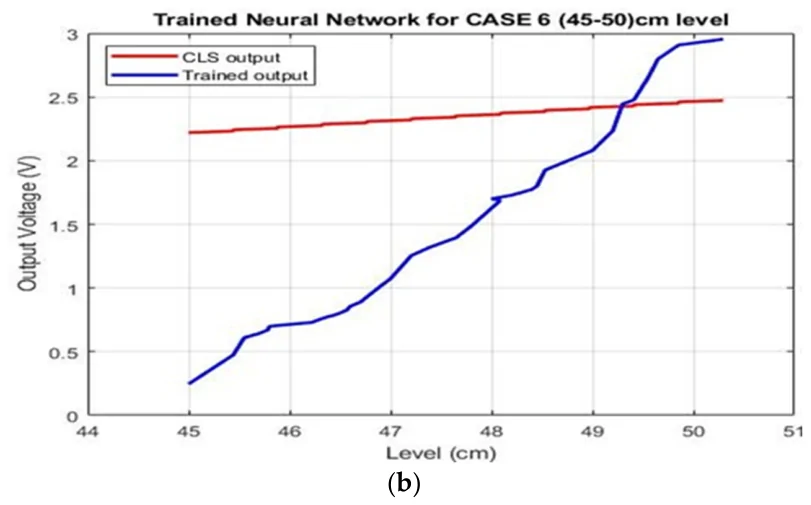
(**a**) Convergences curve of NN. (**b**) Case 6: Output of trained and untrained networks.

Case 6: works for a set range of 45–50 cm. The network is trained to obtain an R value of 0.99918 with an MSE less than 0.00879. The convergence curves of the NN and outputs of the trained network and untrained sensor are shown in Figure 19.

Training and convergence analysis: The convergence of the proposed neural network models was evaluated by monitoring the mean square error (MSE) of the training, validation, and testing datasets with respect to the training epochs. During optimization, the MSE decreases as the network weights and biases are iteratively adjusted using the Levenberg–Marquardt backpropagation algorithm. The convergence criterion is determined from the validation performance rather than from the training loss alone, thereby preventing excessive fitting of the training samples. Training is terminated when further reduction in the training error does not result in a corresponding improvement in validation performance. The convergence plots demonstrate the loss-minimization behaviour of the network and provide a direct comparison between training and generalization performance. The consistently low MSE values and high regression coefficients obtained for the six investigated cases indicate that the trained networks accurately reproduce the desired autorange response while maintaining satisfactory generalization performance.

Sensitivity and resolution should be interpreted as distinct performance characteristics. Autoranging increases the conditioned voltage change corresponding to a unit change in the liquid level and can therefore improve the utilization of the available data-acquisition voltage span. However, an increased output slope does not independently demonstrate an improvement in the minimum physically detectable liquid-level change. The practical-level resolution is additionally determined by the ADC resolution, electronic noise, signal drift, sensor repeatability, hysteresis, temperature dependence, and measurement uncertainty. Consequently, the present results demonstrate an increase in effective output sensitivity, whereas any improvement in physical level resolution requires independent experimental verification.

It is observed that, from the output graph in all the cases, the output of the sensor is standardized to the standard range of 0.010939 to 2.953 V. Additionally, the sensitivity is enhanced for smaller ranges. The results of the NN model for Case 1, Case 2, Case 3, Case 4, Case 5, and Case 6 with respect to different ranges of (0–60) cm, (10–20) cm, (0–30) cm, (15–45) cm, (20–60) cm and (45–50) cm are shown in Table 7. The NN model achieves a standard output within the range of 0.010939 to 2.953 V by training the output of the helical CLS for different user-defined ranges.

**Figure 20:**
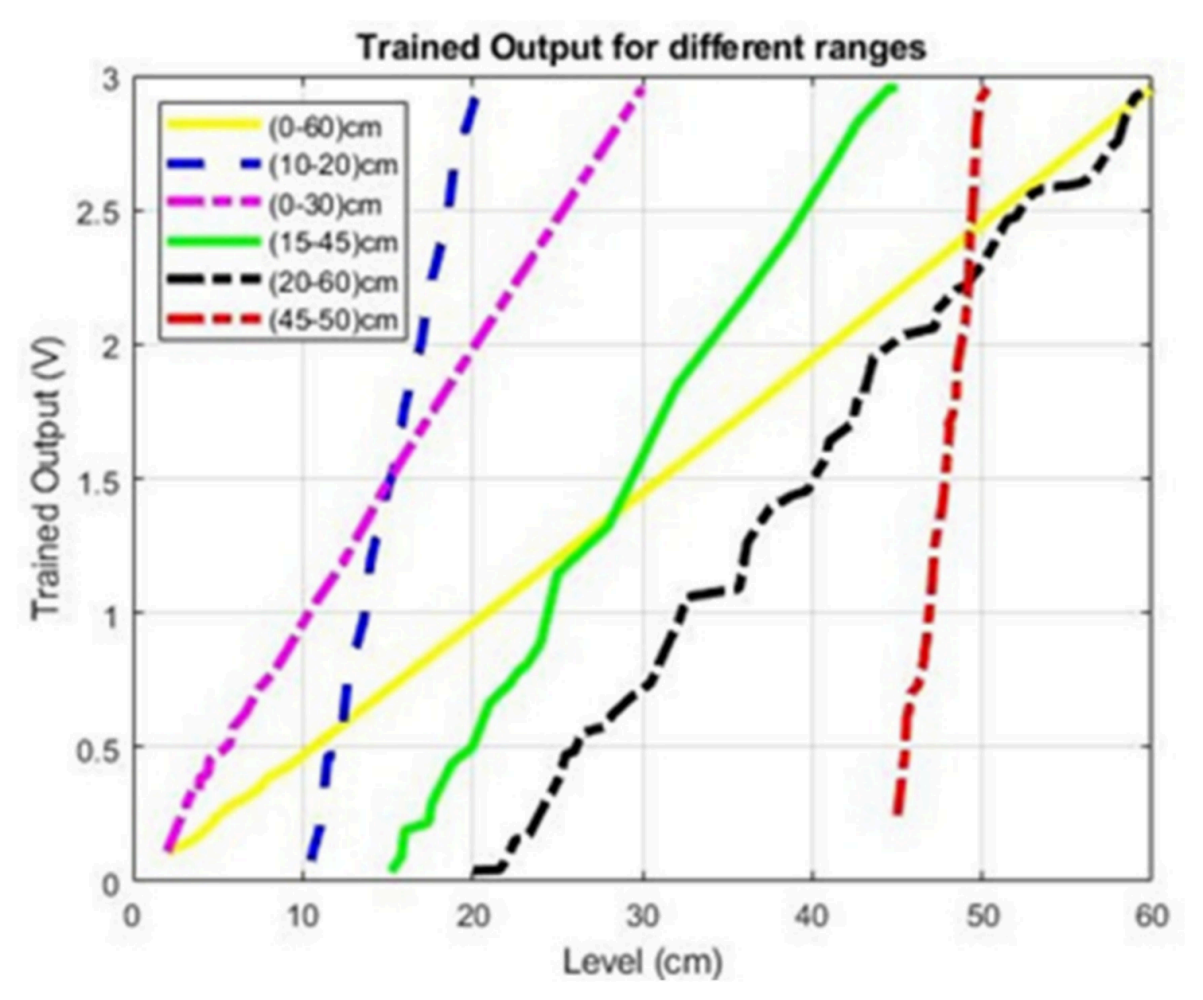
Trained output of the helical CLS.

**Figure 21:**
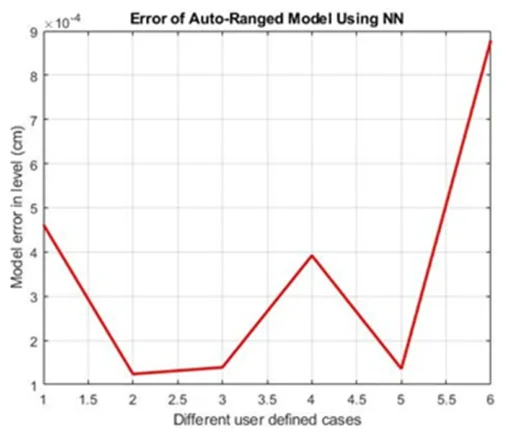
Errors of the trained NN model for different ranges.

Additionally, the trained output plot and error plot of the trained NN model for all the cases with different user-defined ranges with respect to the change in level are shown in Figure 20 and Figure 21. The NN model can enhance the sensitivity for any user-defined range without sensor recalibration. It incorporates the self-calibration technique for any range that is selected by the user, and the results of the enhanced sensitivity are shown in Table 8.

The casewise results further demonstrate the relationship between the selected measurement span and the sensor’s effective sensitivity. For Case 1 (0–60 cm), the complete physical measurement range is used, and consequently, the conventional and autoranged sensitivities remain identical at 4.90429 V/m. For Case 2 (10–20 cm), the sensitivity increases from 4.92253 V/m to 29.42574 V/m, corresponding to an approximately 5.98-fold improvement. For Cases 3 (0–30 cm) and 4 (15–45 cm), the sensitivity reaches 9.80858 V/m, corresponding to approximately 2.02-fold and 1.98-fold improvements, respectively. For Case 5 (20–60 cm), the sensitivity increases from 4.87945 V/m to 7.356435 V/m, representing an approximately 1.51-fold improvement. The most significant increase is obtained for Case 6 (45–50 cm), which has the narrowest measurement span, where the sensitivity increases from 5.03192 V/m to 58.85148 V/m, corresponding to an approximately 11.70-fold improvement. These results demonstrate that reducing the selected physical measurement span while mapping the corresponding sensor response to the complete standardized output-voltage range substantially increases the effective sensitivity.

A combined neural network model is developed to combine all the neural network models of all the different user-defined ranges via Multiplexer (MUX) and loops. The if–else loop statement is added to the model to cater to the selection of cases. All the trained neural networks are combined into a model via MUX. All the cases are incorporated into the model by defining Case 1, Case 2, Case 3, Case 4, Case 5, and Case 6 as 1, 2, 3, 4, 5 and 6, respectively. The user can select any defined range as an input to the model, which results in an output in the standard voltage range, combined with a neural network model in which all the trained networks are included.

The input–output characteristics of the fabricated novel helical electrodes were tested with respect to changes in the liquid concentration. The helical CLS performance was tested, and the CLS output was converted to voltage via signal conversion and conditioning circuits. The results showed that the surface area of CLS increased, which in turn increased the capacitance of the helical CLS. The measured capacitance was 421 µF for 60 cm levels in a tank. The results showed that the CLS output was 0.158 mV from the bridge circuit, and after signal conditioning, the obtained voltage was 2.9564 V. The CLS output from a capacitance of 421 µF was converted to a voltage of 2.9564 V. Additionally, the autorange functionality was developed by training the neural network with the target output by selecting six different set ranges. In Case 1, Case 4, Case 5 and Case 6, at (0–60) cm, (10–20) cm, (0–30) cm, (15–45) cm, (20–60) cm and (45–50) cm, respectively, the network yields a standard output within the range of 0.010939–2.953 V.

In terms of the input–output characteristics, the full-range sensitivity of the helical CLS is 4.904 V/m. The sensitivities obtained by the autorange functionalities for all the set ranges are listed in Table 8. The trained output voltage lies within the standard output range of 0.010939 to 2.953 V. The results show that the sensitivity for smaller ranges is highly enhanced. We can conclude that the autorange functionality enhances the sensitivity of the sensor for different user-defined ranges. This work claims to achieve the objective of this study.

Although the proposed autoranging approach increases the effective voltage sensitivity of the measurement system, sensitivity should not be interpreted independently as a direct gauge of measurement accuracy. The autoranging operation maps the sensor response corresponding to a selected liquid-level interval onto the available standardized voltage range, thereby increasing the voltage variation per unit change in the liquid level. However, this operation does not inherently eliminate the uncertainties originating from the sensing and signal-acquisition chain. The principal uncertainty contributions include reference determination of the liquid level, capacitance measurement, bridge conversion, amplification and signal conditioning, data acquisition, sensor repeatability and hysteresis, and residual prediction error of the neural network model. Consequently, the sensitivity values reported in this study quantify the improvement in the sensor’s electrical response to changes in liquid level and should not be interpreted as an equivalent reduction in overall measurement uncertainty. A complete metrological characterization requires the propagation of individual uncertainty components through the complete measurement chain.

## 6. Conclusions

Liquid-level monitoring is vital for ensuring safety across various process industries. Enhancing the performance of level-measuring instruments in terms of accuracy, range, sensitivity, and precision can significantly improve overall process efficiency. This study introduces an advanced signal conditioning technique designed to enhance the capabilities of liquid-level monitoring systems. This research focuses on developing a signal conversion circuit and implementing a novel autorange functionality to achieve these improvements. This study aims to provide a more reliable and effective solution for liquid-level monitoring in industrial applications through these innovations. The autorange functionality offers several benefits in liquid-level monitoring systems, such as enhanced accuracy, improved sensitivity, a wider measurement range, and increased efficiency.

The sensor output was measured to be 421 µF at a maximum liquid level of 60 cm, which was then converted to a voltage of 2.954 V via De-Sauty’s bridge and a conversion circuit. To increase the sensitivity of the capacitance level sensor (CLS) for any user-defined range, a novel autorange functionality was developed by training neural networks. This technique was implemented and tested on a CLS with a helical structured electrode, 60 cm in height, immersed in a tank 70 cm in height. The sensor’s operating range was determined to be 60 cm, with a full-range sensitivity of 4.904 V/m. The autorange functionality was developed by training neural networks for six different cases: Case 1: 0–60 cm, Case 2: 10–20 cm, Case 3: 0–30 cm, Case 4: 15–45 cm, Case 5: 20–60 cm, and Case 6: 45–50 cm. In each case, the network exhibited a standard output of 0.010939 to 2.953 V. The results demonstrated that incorporating this novel technique significantly enhanced the sensitivity of the level measurement system, resulting in a lower resolution. This work provides a detailed description of the conversion circuit and the innovative autorange technique, which contributes to a more effective and accurate liquid-level measurement system using CLS.

The results should be interpreted by distinguishing between sensitivity enhancement and overall measurement uncertainty. The proposed autoranging method increases the effective voltage variation available per unit change in the liquid level by mapping a selected measurement interval onto the full standardized output range. This enhancement improves the electrical distinguishability of small-level variations within the selected range; however, it does not independently eliminate uncertainty originating from the sensor, reference measurement, capacitance-to-voltage conversion, signal-conditioning electronics, data acquisition system, environmental effects, or neural network prediction. Therefore, the reported sensitivity improvement represents enhanced responsivity of the measurement chain rather than an equivalent reduction in measurement uncertainty. Future development of the system will combine the proposed autoranging approach with higher-precision capacitance-interface electronics and comprehensive uncertainty characterization.

In the future, we will focus on further enhancing the sensitivity of level measurement systems by refining the novel autorange technique. Specifically, investigating the potential for achieving even lower resolution and higher accuracy across a broader range of liquid levels would be valuable. Additionally, exploring the integration of advanced neural network models and adaptive algorithms could lead to more robust and versatile measurement systems. This future research builds on the current work, which successfully described the conversion circuit and autoranging technique, aiming to develop an even more precise and reliable liquid-level monitoring system using capacitance level sensors (CLSs).

## Figures and Tables

**Table 1:** Techniques for enhancing the sensitivity of CLS.

Reference	Types of Sensors Used	Sensitivity Enhancement Techniques	Inferences
[13]	Microfluidic capacitance level	Scalable method	The range of measurements was 0–40 mm. Sensitivity was enhanced by the scalable method. The water level was 0.1 mm; scaled within specified set range.
[14]	Concave capacitance	Design of electrodes	Concave structure electrodes for improving sensitivity. Repeated calibration required.
[15]	Moisture sensor	Shape of electrode	Sensitivity tuned by adjusting electric flux droplet. Recalibration needed.
[16]	Planar microwave liquid	Embedded channel in a substrate	Sensitivity of the proposed sensor was enhanced with the structural addition of substrate by up to 400%; improvement for only the low permittivity materials.
[17]	Dielectric	Structural addition	Higher sensitivity was achieved by loading a microstrip line with multiple coupled resonators.
[18]	Core fibre humidity	Addition of core fibre	Sensitivity of the sensor was enhanced by sandwiching hollow-core fibre between two single-mode fibres.
[19]	Capacitive-level sensor	Spacing of electrodes	Spacing between electrodes and material composition between electrodes is adjusted to provide higher resolution and sensitivity.

**Table 2:** Measured signal values of the helical CLS.

Sl. No	Level	Capacitance	Bridge Output	Conditioned Output
1	Empty Tank (0 cm)	0.929 nF	1.18 mV	0.3 V
2	Full Tank (60 cm)	421 µF	0.158 mV	2.953 V

**Table 3:** Sensor characteristics of helical CLS.

Parameter	Value
Sensor active height (Range)	60 cm
Tank height	70 cm
Level measurement range	0–60 cm
Calibration interval threshold	1 cm
Reported sensor resolution	0.1 cm
Maximum measured capacitance	421 µF
Test liquid	Water
Liquid temperature	25 Deg C
Bridge excitation voltage	5 V
DAQ	NI ELVIS
Sensitivity (capacitance output)	7016 (nF/cm)
Sensitivity (voltage output)	4.90429 (V/m) or 0.0490429 (V/cm)
Hysteresis	0.5 cm

**Table 4:** Sensitivity of the helical CLS for different set ranges.

Cases	Range (cm)	Level (cm)	Output Voltage (V)	Sensitivity (V/m)
Case 1	0–60	0	0.010939	4.90429
		60	2.953513	
Case 2	10–20	10	0.492253	4.92253
		20	0.984506	
Case 3	0–30	0	0.010939	4.84960
		30	1.46582	
Case 4	15–45	15	0.721971	4.958993
		45	2.209669	
Case 5	20–60	20	1.0017323	4.87945
		60	2.953513	
Case 6	45–50	45	2.220608	4.87945
		50	2.472204	

**Table 5:** Specifications of helical CLS.

Sl. No	Specifications	Values
1	Operating Range	(0–60) (cm)
2	Output Voltage	(0.3–2.953) V
3	Resolution	0.1 cm

**Table 6:** Specifications of the neural network model.

Network Parameter	Selected Description
Network Architecture	Feed-Forward Backpropagation
Training Algorithm	Levenberg–Marquardt
Performance Function	Mean Square Error (MSE)
Hidden Layer Activation Function	Tansig
Number of Hidden Layers	2
Neurons in Each Hidden Layer	10
Data Allocation—Training	70%
Data Allocation—Validation	15%
Data Allocation—Testing	15%
Maximum Number of Epochs	1000
Maximum Validation Failures	6
Minimum Performance Gradient	1 × 10^−7^
Initial μ	0.001
μ Decrease Factor	0.1
μ Increase Factor	10
Maximum μ	1 × 10^10^
Performance Goal	0
Model-Performance Indicators	MSE and Regression Coefficient (R)

**Table 7:** Conventional, automatically ranged output and model error for different cases.

Cases	Range (cm)	Level (cm)	Output Voltage of Conventional Model (V)	Output Voltage of Autoranged Model (V)	Sensitivity (V/m)
Case 1	0–60	0	0.010939	0.010939	0.000462
		60	2.953513	2.953513	
Case 2	10–20	10	0.492253	0.010939	0.000124
		20	0.984506	2.953513	
Case 3	0–30	0	0.010939	0.010939	0.000139
		30	1.46582	2.953513	
Case 4	15–45	15	0.721971	0.010939	0.000392
		45	2.209669	2.953513	
Case 5	20–60	20	1.0017323	0.010939	0.000136
		60	2.953513	2.953513	
Case 6	45–50	45	2.220608	0.010939	0.000879
		50	2.472204	2.953513	

**Table 8:** Conventional and autorange full-scale sensitivity of helical CLS for different cases.

Cases	Range (cm)	Sensitivity of Conventional Model (V/m)	Sensitivity of Autoranged Model (V/m)
Case 1	0–60	4.90429	4.90429
Case 2	10–20	4.92253	29.42574
Case 3	0–30	4.84960	9.80858
Case 4	15–45	4.958993	9.80858
Case 5	20–60	4.87945	7.356435
Case 6	45–50	5.03192	58.85148

## Data Availability

This article includes all the data generated or analysed during this study.
